# Effects of home-based neurostimulation on outcomes after stroke: a systematic review and meta-analysis

**DOI:** 10.1007/s10072-024-07633-2

**Published:** 2024-06-28

**Authors:** Auwal Abdullahi, Thomson W. L. Wong, Shamay S. M. Ng

**Affiliations:** 1 Formerly, Department of Rehabilitation Sciences, The Hong Kong Polytechnic University, Hong Kong Special Administrative Region, Hong Kong, China; 2 Department of Rehabilitation Sciences, The Hong Kong Polytechnic University, Hong Kong Special Administrative Region, Hong Kong, China

**Keywords:** Stroke, **Neurostimulation**, Telerehabilitation, Patient-centered care, Activities of daily living, Quality of life

## Abstract

**Background:**

Home-based rehabilitation is a cost-effective means of making services available for patients. The aim of this study is to determine the evidence in the literature on the effects of home-based **neurostimulation** in patients with stroke.

**Method:**

We searched PubMED, Embase, Web of Science, Scopus, and CENTRAL for randomized controlled trials on the subject matter using keywords such as stroke, electrical stimulation and transcranial direct current stimulation. Information on participants’ characteristics and mean scores on the outcomes of interest were extracted. Risks of bias and methodological quality of the included studies were assessed using Cochrane Risks of bias tool and PEDro scale respectively. The data was analyzed using both narrative and quantitative syntheses. In the quantitative synthesis, meta-analysis was carried out using random effect model analysis.

**Result:**

The results showed that, home-based **neurostimulation** is superior to the control at improving upper limb muscle strength (SMD = 0.72, 95% CI = 0.08 to 1.32, *p* = 0.03), functional mobility (SMD = -0.39, 95% CI = -0.65 to 0.14, *p* = 0.003) and walking endurance (SMD = 0.33, 95% CI = 0.08 to 0.59, *p* = 0.01) post intervention; and upper limb motor function (SMD = 0.9, 95% CI = 0.10 to 1.70, *p* = 0.03), functional mobility (SMD = -0.30, 95% CI = -0.56 to -0.05, *p* = 0.02) and walking endurance (SMD = 0.33, 95% CI = 0.08 to 0.59, *p* = 0.01) at follow-up.

**Conclusions:**

Home-based **neurostimulation** can be used to improve upper and lower limb function after stroke.

**Supplementary Information:**

The online version contains supplementary material available at 10.1007/s10072-024-07633-2.

## Introduction

**Neurostimulation** is fast growing in the field of neurological rehabilitation, where many types of patients such as those with stroke, Parkinson’s disease and multiple sclerosis are benefitting from it [1–10]. It is defined as the use of electric, electromagnetic, chemical or optogenetic methods to stimulate or block the flow of action potential through the central nervous system (CNS) [11–15]. In patients with stroke, it is used to help with recovery of brain functions such as sensory, motor and cognitive functions [1, 16].

There are basically two methods of application of **neurostimulation**, invasive (where the stimulation is achieved by surgically implanting electrodes in the stimulation sites) and non-invasive (where the stimulation is achieved by connecting electrodes to the external parts of the stimulation sites such as the skin) techniques. The invasive type of **neurostimulation** includes techniques such as the invasive vagus nerve stimulation (VNS) and deep brain stimulation [17, 18]; whereas, the non-invasive type of **neurostimulation** includes techniques such as the transcutaneous electrical nerve stimulation (TENS), neuromuscular electrical stimulation, orthosis-supported neuromuscular electrical stimulation, transcranial direct current stimulation (tDCS), transcranial alternating current simulation (tACS), transcranial pulse simulation (tPS), transcranial random noise stimulation (tRNS), transcranial magnetic stimulation (TMS), radio-electric asymmetry conveyer (REAC) and non-invasive VNS [19–26]. However, functional electrical stimulation can be used as either non-invasive or invasive type of **neurostimulation** [10].

**Neurostimulation** techniques can be delivered in the clinic or at home [27–29]. A home-based mode of rehabilitation is a healthcare delivery model employed to enhance easy access of rehabilitation services for patients with various conditions [30–33]. Its sole aims are to help reduce the cost of healthcare, and improve patients’ confidence and motivation, and compliance with the rehabilitation [34, 35]. This is because aside from the effectiveness of an intervention based on behavioural and neurophysiological outcomes, its cost is equally important; and a recent suggestion seeks for the use of the most cost-effective interventions [36]. In addition, home-based rehabilitation seems to offer more opportunity for increased intensity of rehabilitation, which is an important factor for recovery of function after stroke [37]. Similarly, it affords the patients with the opportunity to save money on transport, and reduce or prevent the risk of hospital-acquired infections and other communicable diseases, especially during epidemics or pandemics [38–40].

Furthermore, what is very interesting in stroke rehabilitation is that, home-based rehabilitation using exercises produces similar positive results as clinic-based rehabilitation [41]. The aim of this study is to carry out a systematic review and meta-analysis to determine from the literature, the effects of home-based **neurostimulation** in patients with stroke. In addition, the study is aimed at investigating its reported feasibility by summarizing reports of serious adverse events, and participants’ compliance with the protocols.

## Materials and methods

We conducted a systematic review and meta-analysis, which was registered in PROSPERO database (registration number, CRD42023401257) using the Preferred Reporting Items for Systematic Reviews and Meta-Analyses (PRISMA) guideline.

### Inclusion and exclusion criteria

In the study, only randomized controlled trials (RCT) that compared the effects of home-based **neurostimulation** with sham **neurostimulation** or a control intervention on outcomes such as upper limb function, lower limb function, neurophysiological changes, spasticity and adverse events after stroke were included. The studies must also include participants who were 18 years old or more. However, studies that were not published in English language were excluded.

To make effective syntheses of the included studies, they were grouped based on the body part treated (upper and lower limbs) and the outcomes they assessed.

### Procedure for literature search

The following databases: PubMED, Embase, Web of Science, Scopus, and CENTRAL were searched from their earliest dates until July, 2023. In addition, manual search of the references of the included studies and relevant systematic reviews was also carried out [29, 42]. The search was carried out using strategies adapted to the particular database by one of the researchers (AA); however, it was independently verified by another researcher (TWLW). The search terms used include stroke, brain infarction, cerebrovascular accident, electrical stimulation, transcutaneous electrical nerve stimulation, transcranial direct current stimulation, transcranial magnetic stimulation, deep brain stimulation, transcranial alternating current stimulation, transcranial random noise stimulation, telerehabilitation, virtual rehabilitation and remote rehabilitation. See Appendix 1 for the details of the search strategy used.

### Selection of studies and extraction of data

Eligible studies were selected manually and by using Endnote software. The selection was carried out independently by two of the researchers (AA & TWLW).

At first, some of the studies that were ineligible based on the information from their titles and abstract were excluded. However, when the information in their titles and abstract was not sufficient to decide on their eligibility, their full texts were read to decide for their inclusion or otherwise. Moreover, in case of disagreement on the selection decisions between the two researchers (AA & TWLW), a third researcher (SSMN) was consulted for consensus.

Similarly, the data was extracted by one of the researchers (AA); however, it was verified by the other two researchers (TWLW & SSMN). The data extracted include information on the sociodemographic and clinical characteristics of the study participants such as the study authors, participants mean age, time since stroke, sample size, type of stroke and side affected; and the mean scores on the outcomes of interest (primary and secondary outcomes).

The primary outcomes are upper limb function (level of motor impairment, motor function, real world arm use and manual dexterity), lower limb function (walking speed, walking endurance, number of steps, cadence and functional mobility), neurophysiological changes such cortical activation or electrical activity of the muscles, muscle strength, trunk impairment, muscle thickness, spasticity, balance, range of motion, disability and cognitive function. The secondary outcomes are adverse events and caregiver stress.

Since we extracted sufficient information from the studies, no additional information was sought from the authors of the included studies. However, in case of any missing or unreported data, it was designated as ‘not reported.’

### Risks of *bias* and methodological quality assessments

We used Cochrane Risks of Bias Assessment tool and PEDro scale to assess the risks of bias and methodological quality of the included studies. Both the tool and the scale are known to be valid and reliable [43, 44].

The Cochrane Risk of Bias Assessment tool assesses bias due to the selection of participants, blinding of participants and personnel and outcome assessors, attrition and reporting. The result of the assessment is presented in a risk of bias graph.

The PEDro scale consists of 11 items that assess external and internal validity of a study. The external validity is assessed using the first item; whereas, the internal validity is assessed using the remaining 10 items [44]. In addition, a two-point scale, 0 (no) to 1 (yes) is used to rate the responses to the items that assess the internal validity. In this regard, since the scale has 10 items, the possible scores for methodological quality of a study will range between 0 and 10. When the total score ranges between zero and three; or four and five; or six and ten, the methodological quality is said to be low, moderate or high respectively. [45–47] The result of the assessment is presented in a table.

All assessments were carried out independently by two of the researchers (AA & TWLW); however, any disagreements arising from the assessments were managed by consulting the other researcher (SSMN).

### Qualitative and quantitative syntheses of the extracted data

In the qualitative synthesis, a summary of the characteristics, risk of bias and methodological quality, and findings of the included studies was carried out. In the quantitative synthesis, a random effect model meta-analysis was carried out.

In the meta-analysis, the data used were the study sample size, the group mean and standard deviation of the scores on the outcomes of interest at post intervention and follow-up. However, when a study provided median scores and interquartile range on the outcomes of interest, the formulae, mean =$\frac{a+2m+b}{4}$ [where a = the smallest value (minimum), b = the largest value (maximum), and m = median]; and standard deviation = $\frac{IQR}{4}$[where IQR = interquartile range] were used to determine the mean and the standard deviation respectively [48]. Furthermore, percentage of variation across the studies due to heterogeneity (*I*^2^) was deemed significant when the value is between 50 and 90% at *p* < 0.05.

Eligibility for inclusion in the syntheses was determined using a table of characteristics of the included studies to check which studies assessed similar outcomes. The meta-analysis was carried out using RevMan version 5.4.1; [49] and all the results of the meta-analyses were visually displayed using forest plots. In addition, sensitivity analyses of the findings of the included studies were carried out based on the period of the outcomes’ assessments (post intervention and follow-up). In addition, an adapted body of evidence matrix of the Australian National Health and Medical Research Council's (NHMRC) was used to interpret the findings of the study [50].

## Result

### The qualitative synthesis

#### Selection of the studies

The search provided a total of 11,380 studies. Following screening of the studies, only 14 studies were eligible for inclusion [51–64]. However, two other studies seemed to be eligible for inclusion, but they were excluded following careful scrutiny. [65, 66]

**Fig. 1:**
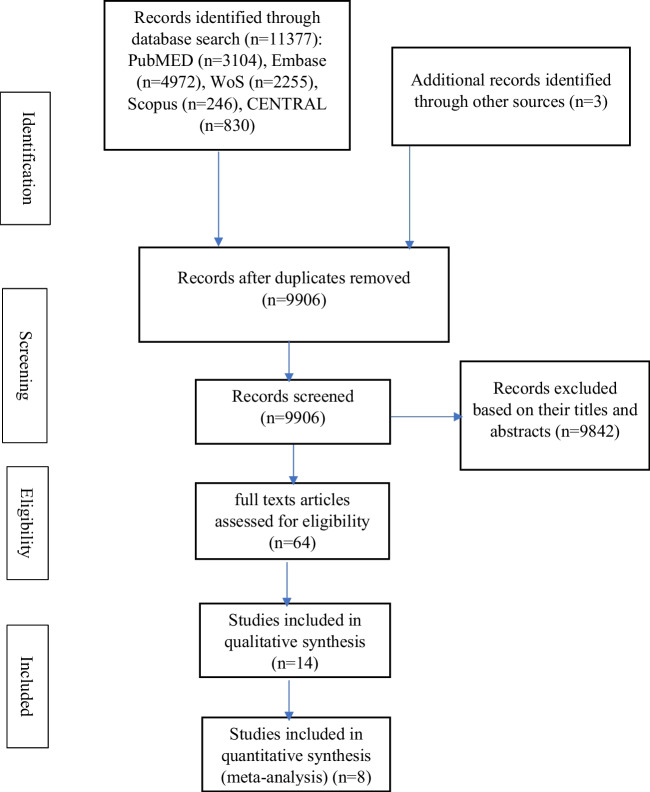
The study flowchart showing the process of selection of the included studies

Among the included studies, two of them contain two experimental and two control groups each [54, 55]; whereas, in one study, there were two experimental groups and one control group [52]. Figure 1 provides the details of the literature search and the process of selection of the eligible studies.

#### Characteristics of the included studies

In total, the number of participants in the included studies was 558. In addition, although two studies did not provide information on sex [51, 55]; 291 and 139 of the participants in the included studies are men and women respectively. Furthermore, only one study included participants in the acute and subacute stages of stroke [59]; all the other studies included participants who were in the chronic stage.

In addition, information on types of stroke was provided in only seven studies [51, 52, 57, 59–61, 63]. In these studies, 212 and 63 participants had ischaemic and haemorraghic stroke respectively. Furthermore, only nine studies provided information on the side affected [53–55, 57, 58, 60, 61, 63, 64]. In these studies, there were 204 and 136 participants who had left and right sided hemiplegia respectively. Similarly, only two studies provided information on handedness before the stroke, wherein one and 43 participants were left and right handed respectively [57, 61].

The inclusion criteria used in the studies include mild to moderate impairment in motor ability [51–53, 55–59, 61, 62, 64]; ability to walk several meters independently [51, 54, 57]; no joint deformity [51]; tolerance for electrical stimulation [51]; impaired sitting balance [60]; no significant cognitive impairment [54–57, 59, 60, 62, 64]; and no significant spasticity [54, 57].

The exclusion criteria used in the studies include presence of severe joint deformity [57, 62, 64]; a debilitating medical or any chronic condition [51, 52, 54–61, 64]; use of chemotherapy, use of anti-spasticity medication or a medication that can impair neuromuscular performance [51, 58, 64]; pregnancy or lactation [59, 64]; having a pacemaker or other implants [51, 56, 57, 59, 60, 62, 64, 67]; excessive pain [57, 58, 61, 62, 64]; presence of aphasia or dysphasia [52, 54]; having severe sensory deficit or neglect [60]; skin infection [57, 59]; hearing or visual impairment [52]; left-handedness before the stroke [61]; and contraindication to stimulation [58].

The result showed that, home-based **neurostimulation** is feasible and improves outcomes such as level of motor impairment, motor function, real-world arm use, manual dexterity, walking speed and endurance, functional mobility, joint range of motion, cortical activity, cognitive function and spasticity. Further details on the study participants, intervention protocols including intensity for the experimental and the control groups, and the outcomes assessed are presented in Table 1.

**Table 1:** Characteristics of the included studies

References	N	*Stroke duration*	Mean age (years)	Intervention	Outcomes	Findings	Adverse events
Alon et al. [51]	*N*=19; experimental (*n*=10, females=no information); control (*n*=9, females=no information)	(experimental = 4.05±2.9; control =4.3±3.3) years	67 ± 6.8years; (experimental = 62.7±11.3; control =62.6±8.2)	FES Experimental= received self-administered stimulation using multi-segment hybrid orthosis-stimulation system . Upper limb- electrodes were placed to ensure accurate stimulation of extensor digitorum, extensor pollicis brevis, flexor digitorum superficialis, flexor pollicis longus, and thenar muscles groups . Lower limb -3 electrodes were positioned over the peroneus longus and tibialis anterior; while 2 electrodes were placed over the 2 heads of gastrocnemius muscle. An alternating current (AC) was delivered at a carrier frequency of 11 KHz for 60 mins two times per day. Control= performed exercise for the distal and upper limb, and foot-leg starting with 10 mins and progressing to 60 mins. Both groups received functional exercises for 60 mins two times per day Interventions in both groups lasted for 3 months in both groups	Gross motor function (BBT), manual dexterity (JHFT), walking speed (10MWT), cadence (10MWT) and number of steps (10MWT)	All outcomes improved better in the experimental group post intervention.	Two participants in the experimental group experienced a temporary and minor skin irritation that resolved after 2 to 3 days
Kimberly et al. [53]	*N*=16; experimental (*n*=8, females=3); control (*n*=8, females=2)	35.5±25.1 months; (experimental = 68.13±39.62; control =22.00±16.06)	60.1±14.5 years years; (experimental = 62.75±7.36; control =53.75±8.53)	NMES Experimental=subjects were instructed to use the electrical stimulator, which delivers 200 μs rectangular biphasic, constant current at 50 Hz, 6 hours per day, for 10 days over the course of 3 weeks. Control= Sham electrical stimulation for the same period	Gross motor function (BBT), quality and quantity of use of the limb, manual dexterity (JHFT), muscle strength (load cell), finger movement control (tracking), and changes in cortical activation (fMRI).	All functional performance outcomes improved in the experimental group. On the other hand, in the control group, only muscle strength improved. However, there was no significant improvement in finger tracking performance and cortical activation in either group.	Fatigue in one patient
Gabr et al. [64]	*N*=12; experimental (*n*=8, females=4); control (*n*=4, females=1)	52.75 ± 39.82 months; (experimental = 68.13±39.62; control =22.00±16.06)	59.7 ± 8.53 years; (experimental = 62.75±7.36; control =53.75±8.53)	NMES Experiment: Participants used electromyography-triggered neuromuscular stimulation (ETMS) at a pulse width, between 100 and 400 μs to stimulate forearm muscles in addition to home exercise, twice every weekday in 35-min increments during an eight-week period Control: performed home exercises comprising of supination/pronation exercises; flexion and extension of the individual fingers; wrist extension and flexion exercises; elbow flexion and extension exercises; and shoulder adduction and abduction exercises. Participants switched groups after 8 weeks.	Compliance (home-use diary), range of motion (goniometry) level of motor impairment (UEFMA), motor function (ARAT)	Range of motion improved in both groups. However, only stimulation used in the experimental group improved level of motor impairment	Not reported
Ng et al. [54]	*N*=80; Control (*n*=20, females=3); TENS (*n*=19, females=2); Placebo+TRT (*n*=20, females=3); TENS+TRT (*n*=21, females=5);	Control (5.2±2.9 years); TENS 6.2±4.1); Placebo+TRT (4.7±4.1); TENS+TRT (5.0±3.0);	Control (57.3±8.6 years); TENS 56.4±9.1); Placebo+TRT (57.1±7.8); TENS+TRT (58.4±7.1);	TENS There are 4 groups in this study. Two of the groups are considered as experimental groups (TENS and TENS+TRT), and they received 60 minutes of TENS (100 Hz, 0.2-ms square pulses, 2 to 3 times sensory threshold) alone, or followed by 60 minutes of TRT comprising of 4 weightbearing and stepping exercises using wooden blocks of 2.5 or 5 cm in height respectively, 5 days a week for 4 weeks. The other two groups are considered as control (placebo+ TENS and control), which received sham TENS and TRT or no treatment at all respectively for the same period as in the experimental group	Ankle flexor spasticity (composite spasticity scale), peak torques of maximum isometric voluntary contraction of ankle dorsiflexors and plantar flexors (load cell) and gait velocity (GAITRite) Compliance- phone call and logbook	Significant increase in gait velocity, functional mobility and walking endurance in the TENS and TENS+TRT group compared to the other groups	Not reported
Hara et al. [62]	*N*=20; experimental (*n*=10, females=2); control (*n*=10, females=4)	(experimental = 13 months±; control =13 months±)	(experimental = 56.0 years; control =60.5 years)	FES Experimental=received a train of biphasic rectangular electric impulses with a pulse width of 50 ms of functional electrical stimulation (FES) to finger and wrist extensors in addition to standard therapy for 1 hour (starting from 30 ins in the first 5 days) per day for 5 months to finger and wrist extensors. Control= received standard therapy alone for the same period.	Motor impairment (SIAS), ROM (goniometer), spasticity (MAS), gross motor function (10-CMT)), manual dexterity (9-HPT)) and muscle activity (EMG)	Significant improvement in active wrist and finger extension and shoulder flexion in the experimental group compared to the control Spasticity decreased in wrist and finger flexors in the experimental group Significant improvement in level of motor impairment, muscle activity, gross motor function and manual dexterity in the experimental group	Not reported
Ng et al. [55]	*N*=109; Control (*n*=29, females=no information); TENS (*n*=28, females==no information); Placebo+TRT (*n*=27, females==no information); TENS+TRT (*n*=25, females==no information);	4.7±3.4 years	56.6±7.9 years	TENS There are 4 groups in this study. Two of the groups are considered as experimental groups (TENS and TENS+TRT), and they received 60 minutes of TENS (100 Hz, 0.2ms square pulses, at 4 lower limb acupuncture sites recommended in Chinses Medicine Literature alone, or followed by 60 minutes of TRT, respectively, 5 days a week for 4 weeks. The other two groups are considered as control (placebo+ TENS and control), which received sham TENS and TRT or no treatment at all respectively for the same period as in the experimental group	Ankle flexor spasticity (Composite Spasticity Scale), peak torques of maximum isometric voluntary contraction of ankle dorsiflexors and plantarflexors (load cell), gait velocity (GAITRite), walking endurance (6MWT)) and functional mobility (TUG) compliance- phone call and logbook	Significant iincrease in gait velocity, functional mobility and walking endurance in the TENS and TENS+TRT group compared to the other groups.	Not reported
Sullivan et al. [58]	*N*=38; experimental (*n*=18, females=13); control (*n*=20, females=4)	7.2 *± *SD (1–29) (experimental = 7.7 *± *SD (1–29) years±; control =6.6 *± *SD (3–14) years	60.6 *± *SD (37–88) (experimental = 61.6 *± *SD (37–88); control =59.5 *± *SD (41–85)	NMES All subjects were instructed to exercise twice daily for 30 minutes, 5 days/week for four weeks. During practice, subjects in the experimental group received electrical stimulation with the following current parameters: symmetrical biphasic waveform, pulse duration 250 microseconds, amplitude at sensory threshold, frequency 35 Hz, and a duty cycle of 10 seconds ON: 10 seconds OFF The control group received sham electrical stimulation.	Level of motor impairment (UEFMA), motor function (AMAT), real world arm use (MAL), tolerance for Eeectrical stimulation (PTTES), stereognosis (Nottingham stereognosis assessment), quality of life (SIS) and spasticity (Tardieu scale)	There was no significant difference between groups in the outcomes of interest.	Not reported
dos Santos-Fontes et al [61]	*N*=20; experimental (*n*=10, females=5); control (*n*=10, females=4)	(experimental = 3.8 ± 4.5; control =3.3 ± 2.1) years	(experimental = 52.2 ± 11.1; control =59.1 ± 11.1)	NMES Experimental=2 hours, daily biphasic square-wave electrical nerve stimulation at a frequency of 31 Hz, immediately before motor training for 4 weeks Control= sham stimulation, 2 hours daily before motor training for 4 weeks. Both groups performed two blocks of the following tasks: writing, turning cards, picking small objects, picking beans with a spoon, and stacking checkers daily for four weeks	Feasibility-compliance (weekly phone call) and safety (presence of adverse events), and manual dexterity (JHFT)	Participants in both groups reported good compliance with the stimulation. However, the participants in the experimental had significantly better compliance. Only 1 participant in the control group reported nocebo effect In addition, manual dexterity improved more significantly in the experimental than the control group	Only 1 participant in the control group reported nocebo effect
Chan [60]	*N*=37; TENS+TRTT (*n*=12, females=4); Placebo + TRTT (*n*=13, females=3); control (*n*=12, females=2)	44.2 ± 28.3 months TENS+TRT (43.9 ± 28.4); Placebo + TRT (41.8 ± 28.7); control (47.3 ± 29.8)	57.8 ± 9.4 years TENS+TRT (58.2 ± 10.7); Placebo + TRT (56.3 ± 7.4); control (59.3 ± 10.4)	TENS The experimental group is the TENS+TRTT; while the control groups are two (Placebo + TRTT and control). The experimental group received high-frequency TENS (frequency 100 Hz; pulse width 0.2 ms) to the abdominal muscles simultaneously with the TRTT at home for 60 mins per day, 5 times a week for 6 weeks. under the instruction of a physical therapist. For the two control groups, the placebo-TENS + TRTT received sham TENS+ TRTT; while the control group did receive any active treatment except health education.	The isometric peak trunk flexion torque and extension torque was measured using a Cybex NORM isokinetic dynamometer, dynamic balance (functional reach test), trunk control (trunk impairment scale)	The experimental groups improved significantly better the control in all outcomes of interest at all periods post intervention and at follow-up. However, TENS+TRT group demonstrated greater and earlier improvement than placebo TENS+TRTT group.	Not reported
Chen [59]	*N*=54; experimental (*n*=27, females=9); control (*n*=27, females=12)	Days; (experimental = 24.96 ± 5.62; control =26.85 ± 4.68)	(experimental = 66.52 ± 12.08; control =66.15 ± 12.33)	NMES Experimental (at home- tele-supervised) and control (conventional in the clinic) Both groups received electromyography-triggered neuromuscular stimulation of ECRL and tibialis anterior muscle of hemiplegic side limbs the for 20 minutes, twice in a working day for 12 weeks, a total of 60 sessions. The stimulation parameters used were: stimulus duration for 5 seconds, intermittent time for 2 seconds, pulse width of 0.2 seconds, frequency of 50 Hz, stimulus intensity of between 8 to 45 mA. Both groups also performed physical exercises comprising of Bobath and Neuromuscular facilitation concepts for 1 hour, twice in a working day for 12 weeks, a total of 60 sessions	Disability and ADL (MBI), balance (BBS), caregiver stress (CSI), muscle contraction condition (EMG),	There was no significant difference between groups in all outcomes of interest post intervention and at follow-up.	Not reported
Minami [56]	*N*=8; experimental (*n*=5, females=2); control (*n*=3, females=1)	8.8±5.6 years	63.1±10.9 years (experimental = 64.0± 13.4; control = 61.7± 10.4)	FES All participants received occupational therapy consisting of 40-min sessions that include range of motion training, strength training, and exercise involving occupational activities with the aim of maintaining and recovering daily life Experimental=10–20 min per session, twice per day, at least 3 time a week of purposeful activity-based electrical stimulation therapy (PA-EST) at 36 Hz for 3 months. Control= stretching/ exercise for the same period Cross over took place after the intervention period	Level of motor impairment (UEFMA), real world arm use (MAL), goal attainment (GAS-light), and muscle thickness of the upper limb and abdominal muscles.	Level of motor impairment, motor function, real world arm use and goal attainment improved significantly in the experimental group compared to the control.	None
Choudhury [52]	*N*=95; experimental paired (*n*=32, females=8); ; experimental random (*n*=32, females=7); control (*n*=31, females=13)	months; (experimental paired= 55 (142); experimental random= 43 (94); control =30 (29))	(experimental paired= 51 (12.1); experimental random= 53 (9.9); control =53 (10.6))	TENS There are to experimental groups (paired and random stimulation groups). In the paired stimulation group, each shock was given 12 ms before the click. For the random stimulation group, the click and shock occurred independently at random, with the same interval distribution as in the paired stimulation group. For the experimental groups, electrodes were placed over the forearm extensor muscles for the transcutaneous electrical stimulation using a single 0.15-ms pulse, over 4 weeks for at least 4 h/d at home from the first day of assessment. Control= received standard care	Motor function (ARAT), ROM (goniometer), muscle torque/ strength, spasticity (MAS), grip strength (dynamometer), maximum muscle contraction (a custom device)	Only experimental paired improved motor function post intervention	Not reported
Prathum et al. [57]	*N*=24; experimental (*n*=12, females=4); control (*n*=12, females=4)	15.92 ± 2.06 months (experimental = 16.33 ± 3.30; control = 15.50 ± 2.60)	57.75 ± 2.45 years; (experimental = 56.83 ± 3.58; control = 58.67 ± 3.70)	tDCs Experimental= 1-h home-based exercise after 20-min dual-tDCS at 2-mA, thrice a week for 4 weeks Control= sham 1-h home-based exercise after 20-min dual-tDCS at 2-mA, thrice a week for 4 weeks The exercise comprises of (1) stretching of the elbow flexor, wrist flexor, and shoulder flexor muscles (hold for 2 min/muscle group); (2) active exercise involving elbow extension, shoulder flexion, forearm pronation, and supination (10 times/ set/ direction, 3 sets/direction/session); (3) reach-to-grasp exercise in different directions (50 times/direction, 3 directions/session)	Level of motor impairment (UEFMA & LEFMA), motor function (WMFT), functional mobility (TUG), walking speed (6MWT), lower-limb functional muscle strength (five times sit to stand test), muscle strength (handheld dynamometer), and grip strength (handgrip dynamometer)	Experimental group had significantly better improvement in level of motor impairment and motor function than the control.	Mild tingling, itching, headache and burning sensation in the experimental group.
Ko et al. [63]	*N*=26; experimental (*n*=12, females=8); control (*n*=14, females=6)	(≥6 months after onset)	59.42±11.32 years; (experimental = 61.25±12.85; control = 57.86±10.04)	tDCs Experimental=tDCS (constant current of 2 mA) self -application, 5 d/wk for 4 weeks for 30 minutes per session. Control= sham tDCS (constant current of 2 mA) self-application, 5 d/wk for 4 weeks for 30 minutes per session. Both groups received 30 mins cognitive therapy comprising of various tasks based on memory and attention areas.	Cognitive function (K-MoCA) Dementia (Korean version of the Dementia Rating Scale-2), lexical retrieval abilities and aphasia (Korean-Boston Naming Test), visual attention and task switching (Trail Making Test), determining the ability of an individual to inhibit a response deemed inappropriate (Go/No Go), and verbal fluency (Controlled Oral Word Association Test) Feasibility (completion rate and protocol adherence)	No significant difference between groups in any of the outcomes post intervention and at follow-up. Feasibility: Adherence rate was 98.4%.	No serious adverse effects were detected

Key: *BBT*=box and block test, *JHFT*=Jebsen Taylor hand function test, *10MWT*= ten-meter walk test, *fMRI* =function magnetic resonance imaging, *NMES*=Neuromuscular electrical stimulation, *FES*=Functional electrical stimulation, Key: *UEFMA*=upper extremity Fugl Meyer motor assessment, *ARAT*=Action research arm test, *SIAS*= stroke impairment assessment set, *ROM*=range of motion, *MAS* =modified Ashworth scale, *10-CMT* = Ten-Cup-Moving Test, 9-*HPT*= Nine-Hole-Peg Test, *EMG*=Electromyography, *TRT*=tasks-related training, *NMES*=Neuromuscular electrical stimulation, *TENS*=Transcutaneus electrical stimulation, Key: *TENS*=transcutaneous electrical nerve stimulation, *TRT*=tasks-related training, *6MWT* =six-minute walk test, *TUG* = timed-up and go test, *UEFMA* = upper extremity Fugl Meyer motor assessment, *AMAT* = arm mobility test, *MAL*=motor activity log, *PTTES*= Perceptual Threshold Test – Electrical Stimulation, *ARAT*=Action research arm test, *SIS*= stroke impact scale, *JHFT*= Jebsen Taylor hand function test, *NMES*=Neuromuscular electrical stimulation, *TENS*=Transcutaneus electrical stimulation. Key: *TRTT* =task-related trunk training, *ECRL*= extensor carpi radialis longus, *MBI*=modified Barthel index, *BBS*=Berg balance scale, *MRS*= modified Rankin scale, *CSI*=caregiver strain index, *EMG*=electromyography, *UEFMA* = upper extremity Fugl Meyer motor assessment, *MAL*=motor activity log, *GAS*-light=goal attainment scale-light, *NMES*=Neuromuscular electrical stimulation, *TENS*=Transcutaneus electrical stimulation, *FES*=functional electrical stimulation. Key: *ARAT*=Action research arm test, *ROM*=range of motion, *MAS* =modified Ashworth scale *tDCss*=transcortical direct current stimulation, *UEFMA*= upper extremity Fugl Meyer motor assessment, *LEFMA*= lower extremity Fugl Meyer motor assessment, *WMFT*=Wolf motor function test, *TUG* = timed-up and go test, *6MWT* =six-minute walk test, *TUG* = timed-up and go test, *K-MoCA*=Korean-Montreal cognitive assessment, *TENS*=Transcutaneus electrical stimulation

#### Risks of bias and methodological quality of the included studies

## Risks of bias

Some of the studies have high risk of bias in allocation concealment (selection bias) [51, 53, 61]; blinding of participants and personnel (performance bias) [51–55, 58–62]; blinding of outcome assessment (detection bias) [51]; and incomplete outcome data (attrition bias) [54–58, 62, 63].

**Fig. 2:**
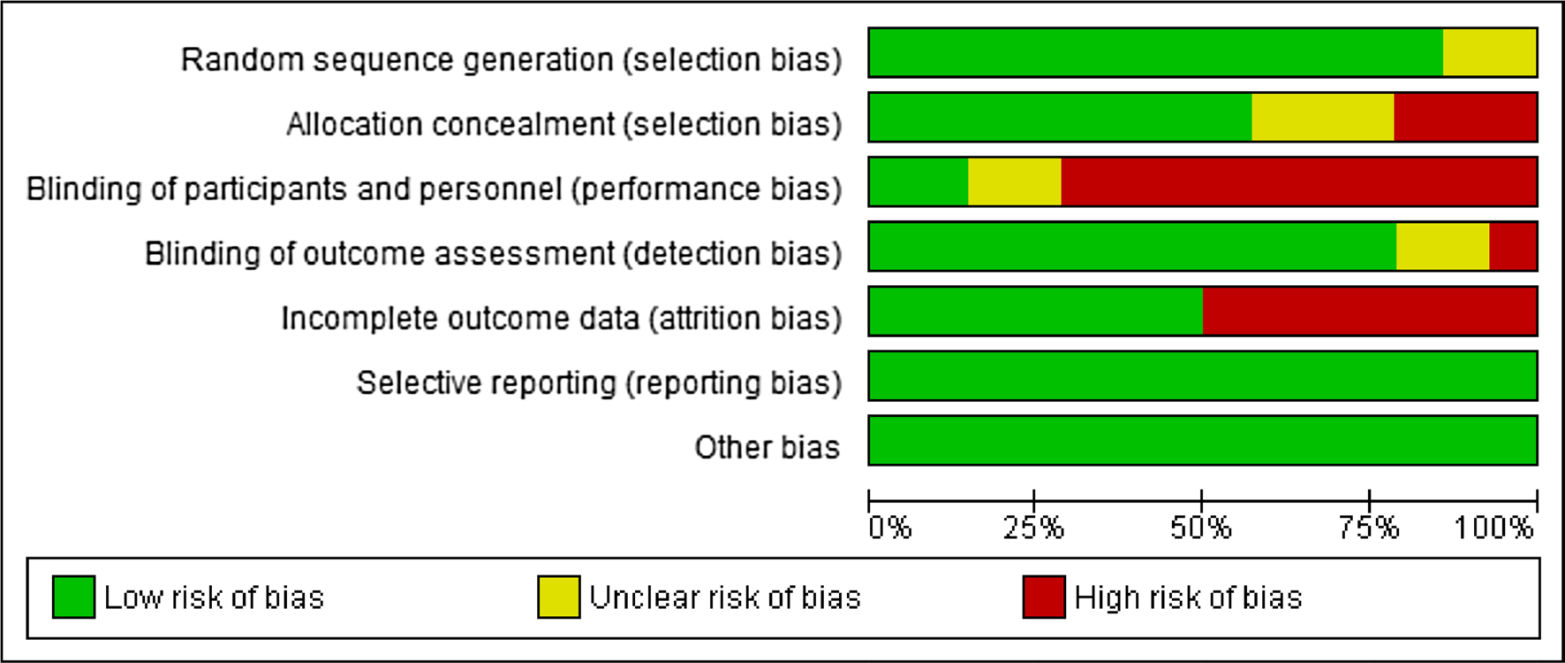
Risks of bias graph of the included studies

Similarly, some of the studies have unclear risks of bias in random sequence generation (selection) [51, 57]; allocation concealment (selection bias [54–56]; blinding of participants and personnel (performance bias) [56, 63]]; and blinding of outcome assessment (detection bias) [56, 63]. See Fig. 2 and Supplementary File 1 for the risk of bias graph and summary table of the included studies respectively.

### Methodological quality

The methodological quality of the included studies is either moderate [56, 63, 64]; or high [51–55, 57–62]. See Table 2 for the met.

**Table 2:** Methodological quality of the included studies

Study	Eligibility criteria specified	Random allocation	Concealed allocation	Comparable subjects	Blind subjects	Blind therapists	Blind assessors	Adequate follow-up	Intention to treat analysis	Between group comparison	Point estimation and variability	Total score
Alon et al. [51]	Yes	1	0	1	0	0	0	1	1	1	1	6/10
Gabr et al. [64]	Yes	1	0	0	0	0	1	1	1	0	0	4/10
Kimberly et al. [53]	Yes	1	0	1	0	0	1	0	1	1	1	8/10
Hara et al. [62]	Yes	1	1	1	0	0	1	1	0	1	1	7/10
Ng et al. [54]	Yes	1	1	1	0	0	1	0	0	1	1	6/10
Sullivan al. [58]	Yes	1	1	1	0	0	1	0	0	1	1	6/110
dos Santos-Fontes [61]	Yes	1	1	1	0	0	1	1	1	1	1	8/10
Ng et al. [55]	Yes	1	1	1	0	0	1	0	0	1	1	6/10
Chan [60]												
Chen [59]	Yes	1	1	1	0	0	1	1	1	1	1	8/10
Minami [56]	Yes	1	0	1	0	0	0	0	0	1	1	4/10
Choudry [52]	Yes	1	1	1	0	0	1	0	1	1	1	7/10
Prathum [57]	Yes	1	1	1	1	0	1	0	1	1	1	8/10
Ko [63]	Yes	1	1	1	0	0	0	0	0	1	1	5/10

### The quantitative synthesis

Only eight studies were used in the meta-analysis for the post intervention outcomes [51–58]. Out of this number, five studies were used for the meta-analysis of upper limb function [52–54, 56–58]; and four studies were used for the meta-analysis of lower limb function [51, 54, 55, 57]. However, for the upper limb, only two studies were included for the meta-analysis at follow-up [52, 57].

For one of the studies, the scores for the outcome of interest were given in median and interquartile range [52]. Consequently, the formulae already explained in the method sections were used to convert them to mean and standard deviation respectively [48].

### Upper limb function

**Fig. 3:**
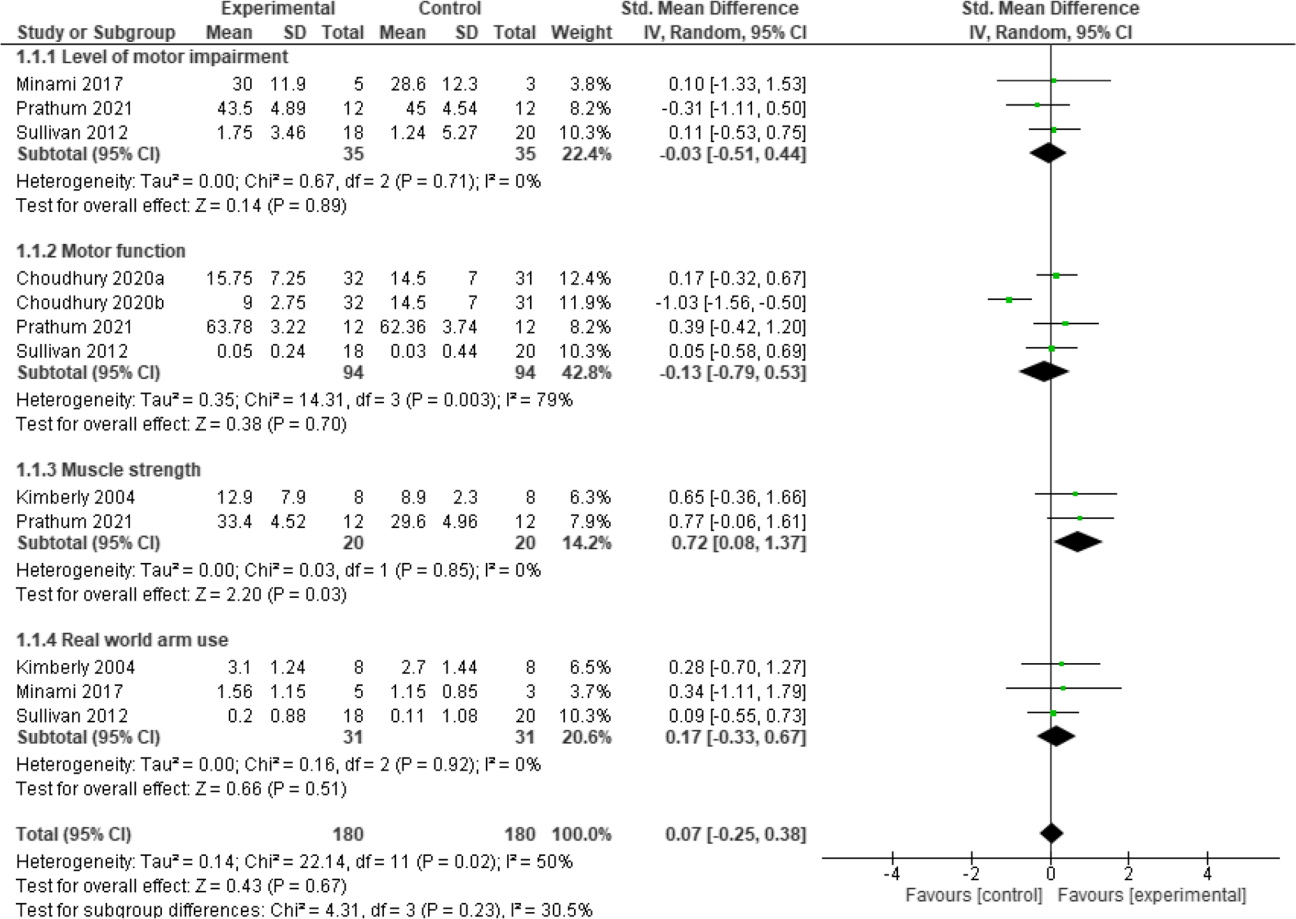
A forest plot showing effects of neuromodulation on upper limb function post intervention

**Fig. 4:**
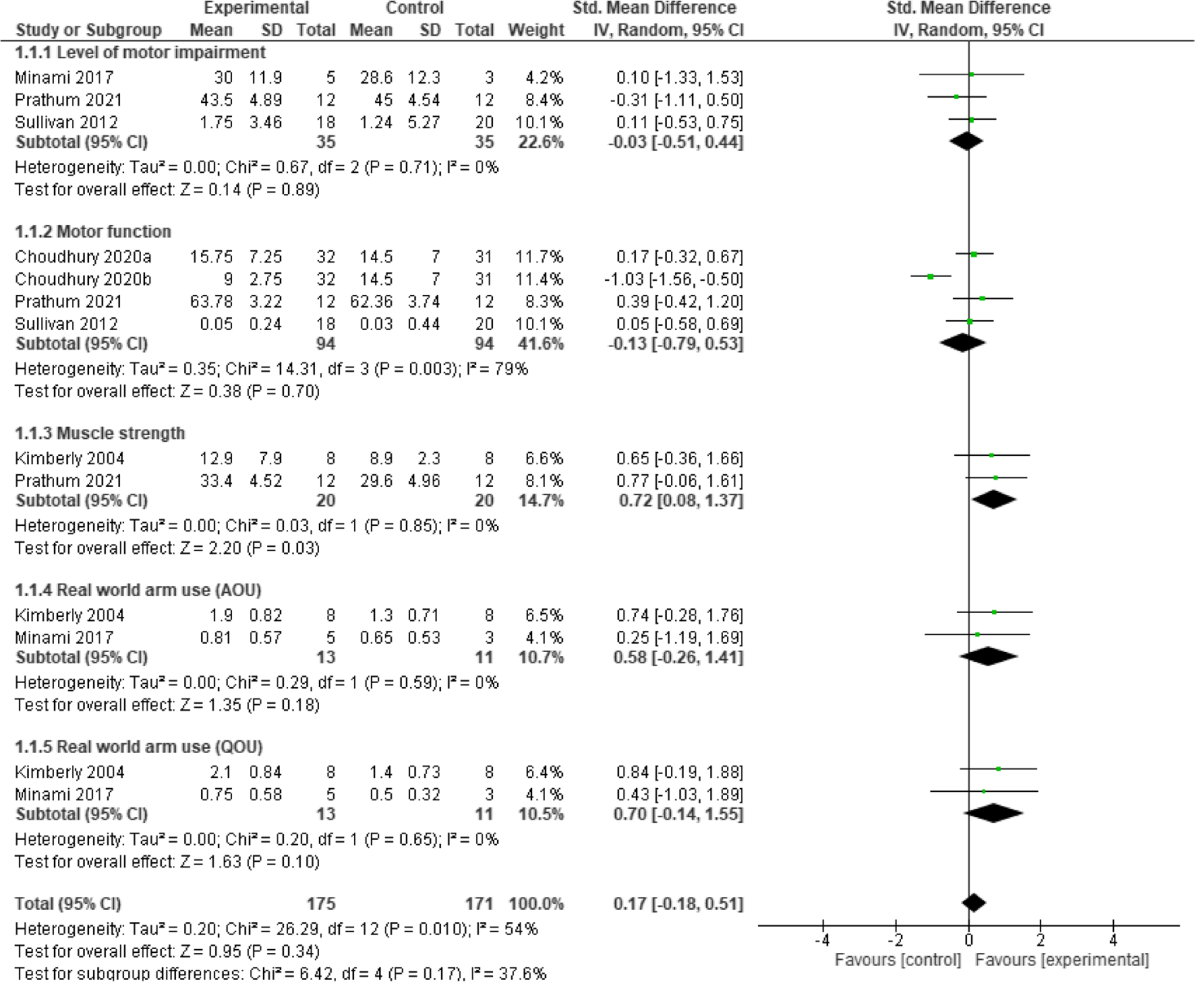
A forest plot showing effects of neuromodulation on upper limb function post intervention (sensitivity analyses)

Post intervention, the result showed that, home-based **neurostimulation** compared to the control, was only superior at improving muscle strength (SMD = 0.72, 95% CI = 0.08 to 1.32, *p* = 0.03). In addition, there was no significant heterogeneity between the included studies (*I*^*2*^ = 0%, *p* = 0.85). See Fig. 3 for the forest plot detailing the result. Furthermore, sensitivity analysis carried out by considering motor activity log (MAL) amount of use (AOU) subscale and MAL quality of movement (QOU) separately, did not reveal any significant difference between groups for the two subscales respectively, (SMD = 0.58, 95% CI = -0.26 to 1.41, *p* = 0.18) and (SMD = 0.70, 95% CI = -0.14 to 1.55, *p* = 0.10). See Fig. 4 for the details of the result.

**Fig. 5:**
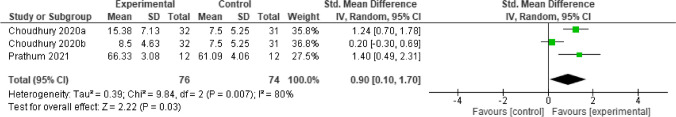
A forest plot showing effects of neuromodulation on upper limb function at follow-up

At follow-up, only two studies assessed one outcome, motor function [52, 57]. The result showed that, home-based **neurostimulation** was superior to the control at improving motor function (SMD = 0.9, 95% CI = 0.10 to 1.70, *p* = 0.03). However, there was significant heterogeneity between the included studies (*I*^*2*^ = 80%, *p* = 0.007). See Fig. 5 for the forest plot detailing the result.

### Lower limb function

**Fig. 6:**
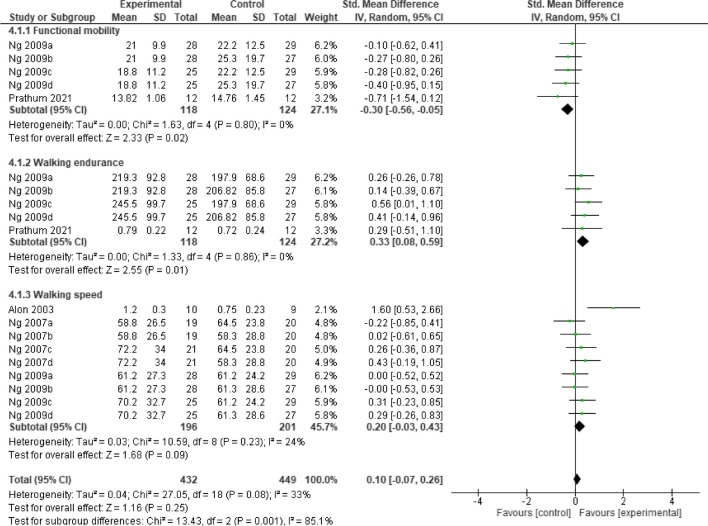
A forest plot showing effects of neuromodulation on lower limb function post intervention

Post intervention, the result showed that, home-based **neurostimulation** compared to the control, was only superior at improving functional mobility (SMD = -0.39, 95% CI = -0.65 to 0.14, *p* = 0.003), and walking endurance (SMD = 0.33, 95% CI = 0.08 to 0.59, *p* = 0.01). In addition, there was no significant heterogeneity between the included studies (*I*^*2*^ = 0%, *p* = 0.49) and (*I*^*2*^ = 0%, *p* = 0.92), respectively. See Fig. 6 for the forest plot detailing the result.

**Fig. 7:**
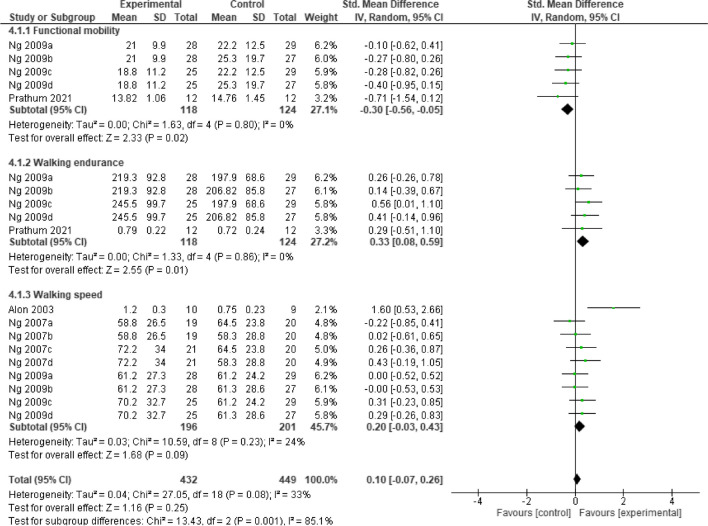
A forest plot showing effects of neuromodulation on lower limb function at follow-up

At follow-up, the result showed that, still home-based **neurostimulation** compared to the control, maintained its superiority at improving functional mobility (SMD = -0.30, 95% CI = -0.56 to -0.05, *p* = 0.02), and walking endurance (SMD = 0.33, 95% CI = 0.08 to 0.59, *p* = 0.01). In addition, there was no significant heterogeneity between the included studies (*I*^*2*^ = 0%, *p* = 0.80) and (*I*^*2*^ = 0%, *p* = 0.86), respectively. See Fig. 7 for the forest plot detailing the result.

### Interpretation of the evidence

It is difficult to be very sure of the evidence since there is variation between studies especially in the use of outcome measures, intensity of rehabilitation used and the types of **neurostimulation** and devices used. However, the evidence seems excellent, appreciably consistent, with satisfactory clinical impact and excellent generalizability and applicability, and as such, it may be used in clinical practice. See Table 3 for more details.

**Table 3:** Body of evidence matrix

Component	Grade	Comments
1. Evidence	A-Excellent Several Level II evidence	Quantity: a total of 14 studies Participants: 558 patients with stroke Level II studies: 14
2. Consistency	C-satisfactory	There is significant heterogeneity between studies, especially in terms of the outcomes assessed and the outcome measures used, devices used for the stimulation and the sample size used
3. Clinical impact	C-Satisfactory	Only one study reported effect size (Prathum et al. [57]
4. Generalizability	A-Excellent	The studied population is the same as the target population (patients with stroke)
5. Applicability	A-Excellent	The evidence is applicable globally since the studies were carried out in 9 different countries (Brazil, China, Japan, Hong Kong (China), Israel, Japan, South Korea, Thailand, and USA) in four different continents
Recommendation	Home-based neuroelectric modulation may be used in practice	

## Discussion

The aim of this study is to determine the effects of home-based **neurostimulation** on outcomes after stroke. The result showed that, home-based **neurostimulation** is feasible and is superior to the control at improving upper limb muscle strength post intervention, and motor function at follow-up. In addition, it is also superior to the control at improving functional mobility and walking endurance both post intervention and at follow-up. This is not surprising since home-based rehabilitation has been reported to be feasible and effective at improving outcomes such as motor function following the use of various interventions such as the constraint induced movement, mirror therapy and therapeutic exercise [29, 68].

Concerning the findings of this study, improvement in muscle strength (an important aspect of motor function), motor function, walking endurance and functional mobility is important for independence in carrying out ADL [69, 70]. For instance, the upper limb is used for eating, washing and grooming oneself. In addition, independence in carrying out ADL is important for overall well-being and good quality of life [71, 72]. Furthermore, it is important for return to work, and by extension economic opportunities and sustainable development [73].

Similarly, impairment in lower limb function may result in sedentary lifestyle and its attendant muscle weakness [74, 75]. Sedentary life is a risk factor for various non-communicable diseases such diabetes, heart disease and depression [76, 77]. Moreover, impaired limb motor function is a significant risk factor for not returning to work after stroke [78]. Thus, finding an intervention such as home-based **neurostimulation** that will help improve the above outcomes and eventually the patients’ quality of life is important. In particular, home-based **neurostimulation**, being a home-based intervention may be more cost-effective and acceptable to patients.

However, home-based **neurostimulation** also has its own limitations like any other home-based rehabilitation. These include problems with the ability of patients and/ or their caregivers to operate the devices and frustration with the use of the devices [79]. In addition, it may be difficult to administer some neurostimulation techniques such as TMS without medical supervision. Furthermore, the cost and size of devices can limit the home-based procedure. However, to help solve some of those problems, we suggest maintaining a regular communication between patients, their caregivers and the clinicians. This can be achieved by using tele-supervision such as via video conference, where the clinicians can observe what the patients are doing [80, 81]. Similarly, community-based rehabilitation can also be used where the clinicians supervise the sessions in person at the patient’s home [82]. In addition, a hybrid model of rehabilitation can be adopted, where in-clinic and home-based sessions are combined to help supplement each other.

Furthermore, the types of **neurostimulation** and the devices used differ between studies. In particular, five out of the eight studies included in the meta-analysis used neuromuscular electrical stimulation [53, 58, 59, 61, 64]; four studies used TENS which is a weaker form of neuromuscular electrical stimulation [52, 54, 55, 60]; three studies used FES [51, 56, 62]; and two used tDCS [57, 63]. These techniques of **neurostimulation** have different mechanisms through which they modulate the nervous system. The neuromuscular electrical stimulation is used to stimulate the peripheral nervous which will indirectly help to modulate the central nervous system (CNS) [16, 83]. The tDCS works to directly modulate the CNS [84, 85]. Thus, the findings of this study may only be limited to the effects of neuromuscular electrical stimulation. However, the findings are still very significant since neuromuscular electrical stimulation is easier to administer compared to other forms of **neurostimulation** such as the tDCS and TMS.

Similarly, in most of the studies, **neurostimulation** was combined with other rehabilitation techniques such as functional exercises. Thus, it is difficult to confidently say the effects were exclusively due to the **neurostimulation**. However, the findings are still a significant milestone since providing rehabilitation at home has so may merits such as the opportunity to increase the intensity of rehabilitation [37]. Therefore, further well controlled studies should be carried out to determine the effects of different forms of home-based **neurostimulation** on outcomes after stroke. In addition, the process of our review is limited in terms of the language in which the included studies were published. Therefore, the findings of the review should be interpreted bearing all the above discussed limitations in mind.

## Conclusion

Home-based neuromuscular electrical stimulation, TENS, FES, and tDCS are feasible and effective at improving many outcomes after stroke. These findings represent a significant milestone since providing rehabilitation at home has so many merits such as the opportunity to increase the intensity of rehabilitation. However, further well controlled studies should be carried out to determine the effects of home-based **neurostimulation** on outcomes after stroke.

## Supplementary Information

Below is the link to the electronic supplementary material.

Supplementary file1 (TIF 54 KB)

## Data Availability

All the data used in this study are included in the manuscript.

## Appendix 1

The Search strategy used in PUBMED, Embase, Web of Science (WoS) and Scopus:

((((((((((((((((((((((((((((Stroke) OR (Ischaemic stroke) OR (Haemorrhagic stroke)) OR (Brain infarction)) OR (Cerebrovascular accident)) AND (Electrical stimulation)) OR (Transcutaneous electrical nerve stimulation)) OR (deep brain stimulation)) OR (Transcranial direct current stimulation)) OR (Anodal Transcranial direct current stimulation)) OR (Cathodal Transcranial direct current stimulation)) OR (Repetitive Transcranial electrical stimulation)) OR (Transcranial electrostimulation)) OR (Transcranial random noise stimulation)) OR (Transcranial alternating current stimulation)) OR (Percutaneous electric nerve stimulation)) OR (Percutaneous electrical nerve stimulation)) OR (Percutaneous electrical neuromodulation)) OR (Percutaneous neuromodulation therapy)) OR (Transcranial magnetic stimulation)) OR (Transcranial magnetic stimulation, repetitive)) OR (Transcranial magnetic stimulation, paired pulse)) OR (Transcranial magnetic stimulation, single pulse)) AND (Telerehabilitation)) OR (Tele-rehabilitation)) OR (Virtual rehabilitation)) OR (Remote rehabilitation)) OR (Telemedicine).

The Search strategy used in CENTRAL:

Stroke AND Electrical stimulation OR Transcranial direct current stimulation OR Transcranial magnetic stimulation AND Telerehabilitation.
