# Correction to: Effects of home‑based neurostimulation on outcomes after stroke: a systematic review and meta‑analysis

**DOI:** 10.1007/s10072-024-07711-5

**Published:** 2024-08-06

**Authors:** Auwal Abdullahi, Thomson W. L. Wong, Shamay S. M. Ng

**Affiliations:** 1 Formerly, Department of Rehabilitation Sciences, The Hong Kong Polytechnic University, Hong Kong Special Administrative Region, Hong Kong, China; 2 Department of Rehabilitation Sciences, Hong Kong Special Administrative Region, The Hong Kong Polytechnic University, Hong Kong, China

**Correction to: Neurological Sciences (2024)**

10.1007/s10072-024-07633-2

The original version of this article unfortunately contained a mistake in Table 1 alignment. The errors pertain to the information under the column ‘Findings’ in page 3 of Table 1.The studies involved are Ng et al. [2009] and Sullivan et al. [2012].

The original article has been corrected.

