# Supplementary figures and images for: Effects of home-based neurostimulation on outcomes after stroke: a systematic review and meta-analysis
Source: Neurol Sci. 2024 Jun 28;45(11):5157–79. doi: 10.1007/s10072-024-07633-2 (PMC11470900; doi:10.1007/s10072-024-07633-2)

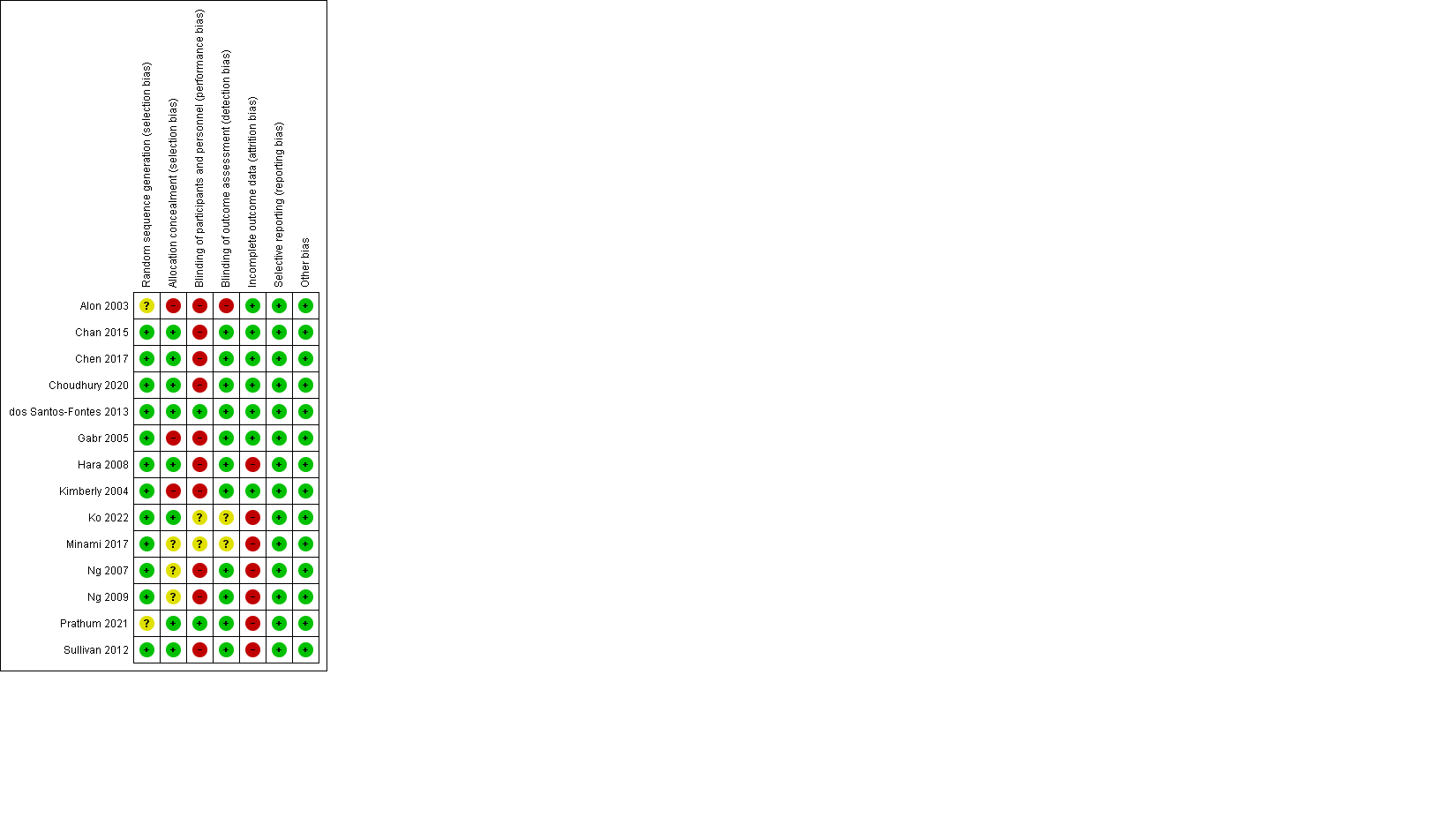

Supplement: Supplementary file 1 — Supplementary file1 (TIF 54 KB) [file 10072_2024_7633_MOESM1_ESM.tif]
